# Supplementary material for: Sustained-input switches for transcription factors and microRNAs are central building blocks of eukaryotic gene circuits
Source: Genome Biol. 2013 Aug 23;14(8):R85. doi: 10.1186/gb-2013-14-8-r85 (PMC4054853; doi:10.1186/gb-2013-14-8-r85)
Supplement: Additional file 5 — HTML Browsable Motif Output. Zipped folder containing all WaRSwap and FANMOD motif output, viewable in a web browser. [file gb-2013-14-8-r85-S5.ZIP › HTML_browsable_motif_output/WaRSwap_all_tair9/sigs_WSWRSLDraw-2000_all.pvals.heatmaps.html/motif_id_14_011110002_tftype_all_upstream_-2000_0.html]

```
BG_MODEL = Rnew2
MOTIF_ID = 14_011110002
TF_TYPE = all
UPSTREAM = -2000_0


PVals
FN_0.2	FN_0.4	FN_0.6	FN_0.8
dg_60.genes	0.253	0.003	0.024	0.689
dg_70.genes	0.195	0.001	0.029	0.666
dg_80.genes	0.63	0.084	0.241	0.559

ZScores
FN_0.2	FN_0.4	FN_0.6	FN_0.8
dg_60.genes	0.698	3.446	2.446	-0.835
dg_70.genes	0.9	3.748	2.525	-0.808
dg_80.genes	-0.428	1.583	0.409	-0.701

StDevs
FN_0.2	FN_0.4	FN_0.6	FN_0.8
dg_60.genes	113231.355	52925.327	15444.154	2461.155
dg_70.genes	112145.703	51051.241	15550.808	2349.771
dg_80.genes	89361.467	42076.095	12769.364	2015.407
```
